# Efficacy of combination triple therapy with vasopressin, steroid, and epinephrine in cardiac arrest: a systematic review and meta-analysis of randomized-controlled trials

**DOI:** 10.1186/s40560-022-00597-5

**Published:** 2022-02-02

**Authors:** Fatemeh Saghafi, Negar Bagheri, Amin Salehi-Abargouei, Adeleh Sahebnasagh

**Affiliations:** 1 Department of Clinical Pharmacy, Faculty of Pharmacy and Pharmaceutical Sciences Research Center, Shahid Sadoughi University of Medical Sciences, Yazd, Iran; 2 Pharmaceutical Sciences Research Center, School of Pharmacy, Student Research Committee, Shahid Sadoughi University of Medical Sciences, Yazd, Iran; 3 Nutrition and Food Security Research Center, Shahid Sadoughi University of Medical Sciences, Yazd, Iran; 4 Department of Nutrition, School of Public Health, Shahid Sadoughi University of Medical Sciences, Yazd, Iran; 5 Clinical Research Center, Department of Internal Medicine, School of Medicine, North Khorasan University of Medical Sciences, Bojnurd, Iran

**Keywords:** Cardiac arrest, Corticosteroid, Vasopressin, Epinephrine, Survival, Return of spontaneous circulation, Triple therapy, Systematic review, Meta-analysis, In-hospital cardiac arrest

## Abstract

**Background:**

This study investigated whether combination therapy with vasopressin, steroid, and epinephrine (VSE) improves in-hospital survival and return of spontaneous circulation (ROSC) during and after resuscitation in-hospital cardiac arrest (CA).

**Materials and methods:**

Various databases were explored from inception until October 2021 for relevant published clinical trials and cohort studies.

**Results:**

Three clinical trials were included. Pooled analysis suggested that VSE was significantly associated with increased ROSC in patients with in-hospital CA (IHCA) (odds ratio (OR): 2.281, 95% confidence interval (CI): 1.304–3.989, *P* value = 0.004). Meta-analysis of two studies (368 patients) demonstrated a significant difference in the reduction of mean arterial pressure (MAP) during and 15–20 min after cardiopulmonary resuscitation (standardized mean difference (SMD): 1.069, 95% CI: 0.851–1.288, *P* value < 0.001), renal failure free days (SMD = 0.590; 95% CI: 0.312–0.869 days; *P* value < 0.001), and coagulation failure free days (SMD = 0.403; 95% CI: 0.128–0.679, *P* value = 0.004). However, no significant difference was observed for survival-to-discharge ratio (OR: 2.082, 95% CI: 0.638–6.796, *P* value = 0.225) and ventilator free days (SMD = 0.201, 95% CI: − 0.677, 1.079 days; *P* value = 0.838).

**Conclusions:**

VSE combination therapy during and after IHCA may have beneficial effects in terms of the ROSC, renal and circulatory failure free days, and MAP.

*Prospero registration*: CRD42020178297 (05/07/2020).

**Supplementary Information:**

The online version contains supplementary material available at 10.1186/s40560-022-00597-5.

## Background

Cardiac arrest (CA) is referred to as sudden loss of blood flow resulting from heart struggle to effectively pump blood to the brain and other vital organs [1]. Some physiological events, such as intense sympathetic stimulation and subsequent vasoconstriction, and the increased heart rate and respiratory drive occur that improve microcirculation, coronary perfusion, and cardiac contractility during CA [2]. Loss of consciousness, hypoxemia, and dyspnea are the main warning signs of this disorder. Some other signs and symptoms may also occur before CA, including chest pain, weakness, tachypnea, fluttering or palpitation; however, sudden CA often takes place with no warning signs. If CA is not treated within the initial minutes, it typically leads to death. The advanced cardiac life support (ACLS) guidelines recommend initiation of adult basic life support (BLS) algorithm and high-quality cardiopulmonary resuscitation (CPR). CPR and early defibrillation are fundamental steps for a successful life support in CA. In case of hypoxemia, supplemental oxygen is also recommended. Pharmacotherapy in CA includes administration of 1 mg of epinephrine every 3–5 min intravenously or intraosseously. If the second defibrillation fails, antiarrhythmic drugs of amiodarone or lidocaine should be initiated [3].

Epinephrine (EP) has been considered a main choice of life support for CA for decades [4]. Epinephrine is an active sympathomimetic hormone, stimulating alpha- (α) and beta (β)-adrenergic systems. EP increases the likelihood of achieving the return of spontaneous circulation (ROSC). However, high circulating catecholamines and overwhelming sympathetic tone are associated with oxidative stress and apoptosis of myocardiocytes. These neurohormonal events eventually lead to multiple organ dysfunction syndrome and are correlated with poor hemodynamic and neurological outcomes [5–7]. Some studies have shown that EP impairs the cerebral microcirculatory flow [8]. Once ROSC is achieved, excessive plasma concentrations of EP cause ventricular tachycardia, subsequent increase in oxygen demand, and ventricular fibrillation [9]. Therefore, attempts have been made to explore further drug combinations to reduce exogenous catecholamines requirements in cardiac arrest.

Vasopressin (VP), a non-adrenergic peripheral vasoconstrictor, causes narrowing of coronary and renal arteries. It has been considered as a therapeutic modality during CPR [10]. In-vitro studies have indicated that adjuvant therapy with exogenous VP during CPR is more effective than optimal doses of EP in improving blood flow to critical organs [11]. VP enhances the arterial and coronary perfusion pressure [12]. Unlike exogenous catecholamines, VP has minimal chronotropic effects and does not increase myocardial oxygen consumption [13]. At lower doses, VP activates the intravascular P2 purinergic and oxytocin receptors (OTR), through which, lower doses of VP mediate endothelial vasodilation, attenuate coronary vasoconstriction caused by V1 vascular receptors (V1Rs),and exert a positive inotropic effect [14, 15]. Moreover, VP stimulates secretion of cortisol directly through V1R and V3R on adrenal cortex [16]. However, the result of a previous systematic review of the randomized clinical trials (RCTs) could not display a benefit for VP, in combination with EP or only VP over EP, in improving survival of out-of-hospital cardiac arrest (OHCA) [17]. Thus, more investigations are needed to clarify the exact role of VP in management of CA [18].

Glucocorticoids (GCs) exert key metabolic influences on carbohydrate, lipid, and protein metabolism and maintenance of electrolyte and fluid balance. GCs increase blood glucose concentrations and accelerate glucose delivery during acute stress [19]. Endothelial glycocalyx is a combination of membrane-bound proteoglycans and glycoproteins covering endothelium luminally. Vascular endothelium is coated in endothelial glycocalyx, reduction of which increases capillary permeability [20]. Overwhelming systemic inflammatory responses contribute to glycocalyx shedding [21]. It has been demonstrated that endothelial glycocalyx plays a key role in pathophysiological events after CA, which is called as post-cardiac arrest syndrome (PCAS). Furthermore, serum levels of endothelial glycocalyx components are raised significantly immediately after CA [22]. Various studies have suggested that glycocalyx is a target for corticosteroids and this class of medications prevents endothelial glycocalyx shedding and diminishes interstitial swelling [23]. Previous studies have shown the adrenal insufficiency during CPR, manifested with a low serum cortisol concentration. Moreover, serum cortisol levels are lower in non-survivors of CA than survivors [24, 25]. Although, there are some evidence stating that GCs may have beneficial effects in CA, they suffer from low quality and fundamental flawed methodology.

Therefore, mechanically, it seems that the combination of these three drugs can be associated with better efficacy in IHCA via improving blood flow to critical organs and stabilization of endothelial glycocalyx barrier. As a result, several clinical trials have been designed to evaluate the effect of pharmacotherapy with the combination of these three drugs of vasopressin, epinephrine and glucocorticoids in CA patients, which have been associated with conflicting results in improving the return of spontaneous circulation during cardiopulmonary resuscitation and patient survival [26–28]. Considering the high mortality rate in CA victims, many attempts have been made to explore some therapeutic strategies to improve survival in CA. Although, the previous studies have assessed the effect of VP, EP, or GCs alone or in combination in CA, there is inconclusive evidence regarding combination therapy with VP, EP, and GCs (VSE) in the management of CA. Therefore, objective of the current study was to systematically review the existing literature on the efficacy of combination therapy with VSE in CA and investigating whether this combination therapy improves survival in victims of both in-hospital cardiac arrest (IHCA) and out of hospital cardiac arrest.

## Methods

### Protocol and registration

This systematic review was designed in accordance with the PRISMA (preferred reporting items for systematic reviews and meta-analyses) statement and it was registered in the International Prospective Register of Systematic Reviews (PROSPERO) on May 2020 (Registration Number: CRD42020178297).

### Data sources and search strategy

The keywords used in the present search strategy were selected from the medical subject headings (MeSH) database and other related non-MeSH terms. MEDLINE in PubMed (www.pubmed.com; National Library of Medicine), Scopus (www.scopus.com), ISI Web of Science (www.thomsonreuters.com), Cochrane central register for controlled trials (https://www.cochranelibrary.com/central/about-central) and Google Scholar (www.scholar.google.com) databases were searched for the following keywords: “Hydrocortisone” OR “Steroid” OR “Glucocorticoids” OR “Methylprednisolone” in combination with “Epinephrine” OR “Adrenaline” OR “EP” in combination with “Vasopressin” OR “Antidiuretic Hormones” in combination with “Cardiac Arrest” OR “Heart Arrest” OR “Sudden Cardiac Death” (Search strategy is presented in Additional file 1: Appendix A).

### Eligibility criteria

Herein, the studies conducted on adult (aged ≥ 18 years) patients with CA (population), who had received VP (20 IU/CPR cycle) plus EP (1 mg/CPR cycle; cycle duration of approximately 3 min) or isotonic saline chloride placebo plus EP (1 mg/CPR cycle) for the first 5 CPR cycles after intervention were considered. Additional EP was administered afterwards if needed. Patients in the intervention group received methylprednisolone sodium succinate (40 mg) and those in the control group received isotonic saline chloride as placebo during the first CPR cycle. Patients with post-resuscitation shock were treated with stress dose of hydrocortisone (intervention and comparison). The selected studies had reported survival-to-discharge ratio, ROSC for 15 to 20 min or longer, and organ failure free days (outcomes).

Studies conducted on the subjects aged under 18 years, patients with terminal illness (life expectancy of 6 weeks), or undergone treatment with steroid within 1 month prior to hospital referral were excluded from the study.

Two investigators (FS and NB) scanned title and abstract carefully to exclude irrelevant papers and then, full text of the remained papers was evaluated. All the duplicated pooled records were removed automatically and manually via EndNote software (version X7).

### Quality of evidence

A modified version of the Cochrane Collaboration’s tool [29–31] was used for assessing risk of bias in RCTs. Random sequence generation (selection bias), allocation concealment (selection bias), blinding of participants and personnel (performance bias), blinding of outcome assessment (detection bias), and incomplete outcome data (attrition bias) were considered to summarize the quality of the selected studies. Each potential source of bias was graded as low, high, or unclear risk. A summary of overall assessment was provided by considering all the mentioned domains.

### Outcomes and prioritization

Primary outcomes were ROSC within 15–20 min or longer. Secondary outcomes included survival-to-discharge ratio, number of organ failure free days, risk of complication and adverse events, neurological status and average mean arterial pressure (MAP) during and 15–20 min after CPR.

### Data extraction

Three reviewers (FS, AS and NB) extracted the data using a standardized form of Cochrane Data Collection for Randomized Controlled Trials, independently. The following information was collected from each of the included studies: The family name of the first author, year of publication, country where the study was implemented, design of the study, baseline demographic and clinical characteristics of the patients, inclusion and exclusion criteria, primary and secondary outcomes, the drugs used in the intervention and control groups, and the intervention period.

### Risk of bias assessment s in individual studies

Risk of bias in each of the included studies was independently assessed by 2 reviewers (NB and FS) using the Cochrane Collaboration risk of bias tool [32]. Six main domains of bias including selection, attrition, detection, performance, reporting bias and the other sources were evaluated using Review Manager software, which were finally classified as ‘low-risk’, ‘high-risk’, or unclear-risk studies. Studies that had a low risk of bias for all domains were regarded to have good quality; studies where one criterion was high risk or two criteria were unclear had fair quality, and the studies were listed as poor-quality studies if they had two or more items with high risk or unclear risk of bias.

### Compliance with ethics guidelines

This systematic review and meta-analysis was carried out on published studies, so it does not involve any human or animal studies performed by the authors of this study. Shahid Sadoughi University of Medical Sciences, Yazd, Iran approved this research.

### Statistical analysis

Standardized mean difference (SMD, Cohen’s *d*) and its corresponding standard error (SE) were calculated for continuous variables to be used as effect size for meta-analyses. For dichotomous variables, odds ratio (ORs) and their corresponding 95% confidence interval were derived and the logarithm of ORs and their corresponding SE were calculated as effect size [33]. Meta-analyses were performed using a random-effects model. Sensitivity analysis was conducted to determine the extent to which summary of the effects might depend on a particular study or a group of publications. In the case of significant asymmetry in funnel plots, trim -and-fill analysis was done to see if the overall effect was changed after establishing symmetry in the funnel plot. All the statistical analyses were done using Comprehensive Meta-Analysis Software (CMA) version 2. *P *values < 0.05 were considered as statistically significant.

## Results

### Description of search

**Fig. 1:**
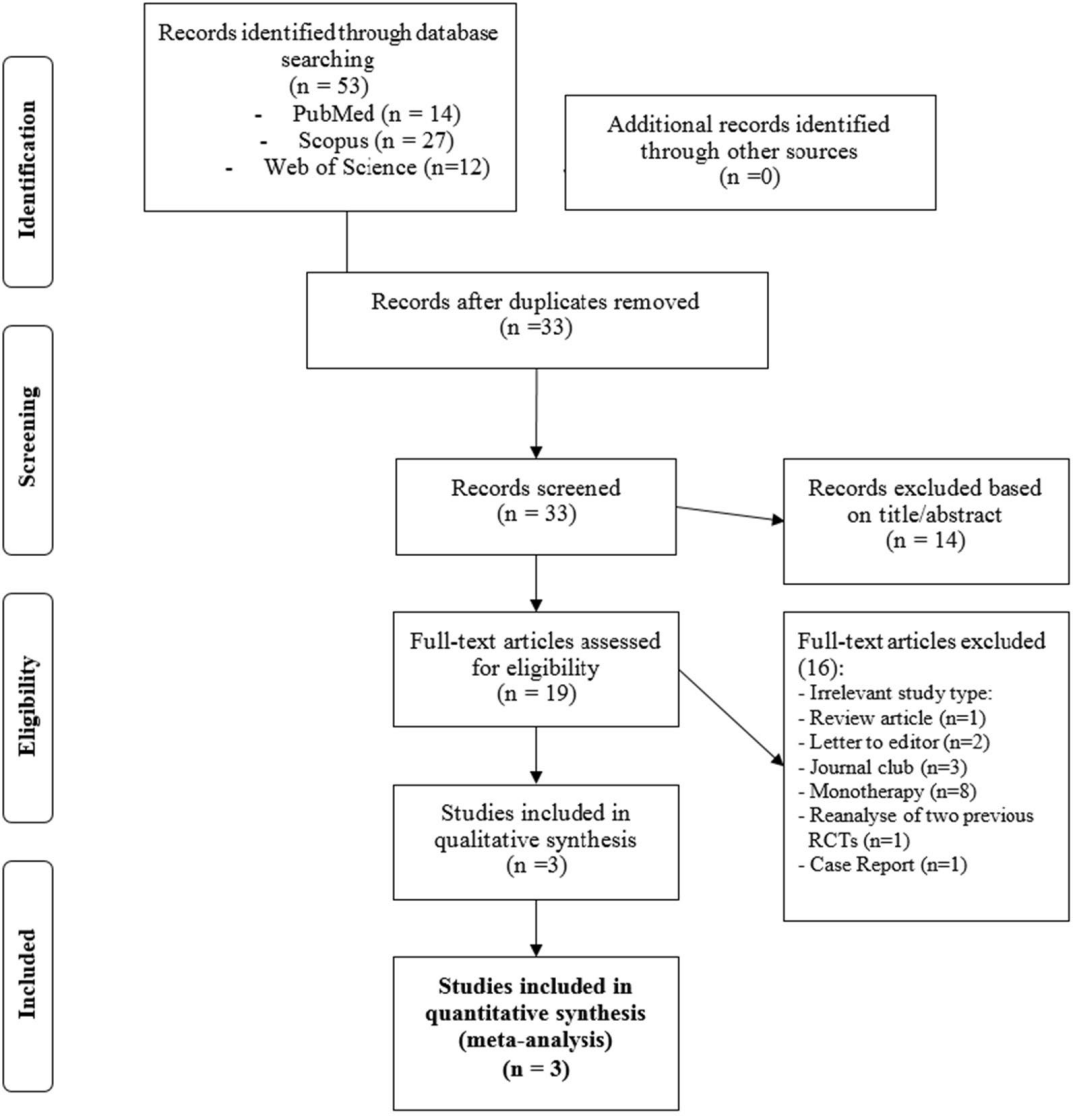
PRISMA flow diagram and study selection process

The primary searches on the previously mentioned databases resulted in identification of 53 papers with some overlaps between PubMed, Web of Science, SCOPUS, and EMBASE databases. No new paper was found by manual searching or searching on databases, such as Google Scholar. After removal of the duplicated papers, only 34 papers remained. Fourteen papers were excluded after screening the titles and abstracts (Tiab). After assessment of full texts of the studies, 17 papers were also excluded from further evaluation, because of irrelevant population and the type of studies. A total of three RCTs were eligible for including in the current systematic review and meta-analysis, carried out on 869 subjects [26–28]. The PRISMA flow diagram for screening inclusion/exclusion criteria in identification of the related papers is illustrated in Fig. 1.

### Characteristics of the included studies

All of the studies were parallel RCTs and were written in English language. Among three RCTs, two studies were conducted in Greece [27, 28] and the other one in Denmark [26].

All the studies were published during 2009—2021. Duration of the studies varied between 10 and 30 months. The characteristics of the included studies are presented in Table 1.

**Table 1:** Characteristics of included studies of glucocorticoids in cardiac arrest

Study details	Mentzelopoulos et al. [28]	Mentzelopoulos et al. [27]	Andersen et al. [26]
Country	Greece	Greece	Denmark
Period of enrollment	2005 to 2006	2008 to 2010	2018 to 2021
Type of population			
In-hospital	**✓**	**✓**	**✓**
Sample size, *N*	100	268	501
Cases for analysis, *N*			
VSE group	48	130	237
Control group	52	138	264
Age, y			
VSE group	65.5 ± 17.7	63.2 ± 17.6	71.0 ± 13.0
Control group	69.2 ± 17.7	62.8 ± 18.6	70.0 ± 12.0
Male gender, %			
VSE group	63	73.1	62
Control group	56	63.8	66
Study design			
Prospective RCTs	**✓**	**✓**	**✓**
Inclusion and exclusion criteria defined	**✓**	**✓**	**✓**
Excluded patients specified	**✓**	**✓**	**✓**
Relevant baseline characteristics	**✓**	**✓**	**✓**
Reporting			
ROSC	**✓**	**✓**	**✓**
Survival	**✓**	**✓**	**✓**
MAP during and after CPR	**✓**	**✓**	
Ventilator free days	**✓**	**✓**	**✓**
Organ failure free days	**✓**	**✓**	
Complication and Adverse events	**✓**	**✓**	**✓**
Neurologic Outcome	**✓**	**✓**	**✓**

Values are mean ± SD where appropriate

*N* number, *VSE* vasopressin, steroid, and epinephrine, *RCTs* randomized clinical trials, *ROSC* return of spontaneous circulation, *MAP* mean arterial pressure, *CPR* cardiopulmonary resuscitation

### Risk of bias in the reviewed studies

**Fig. 2:**
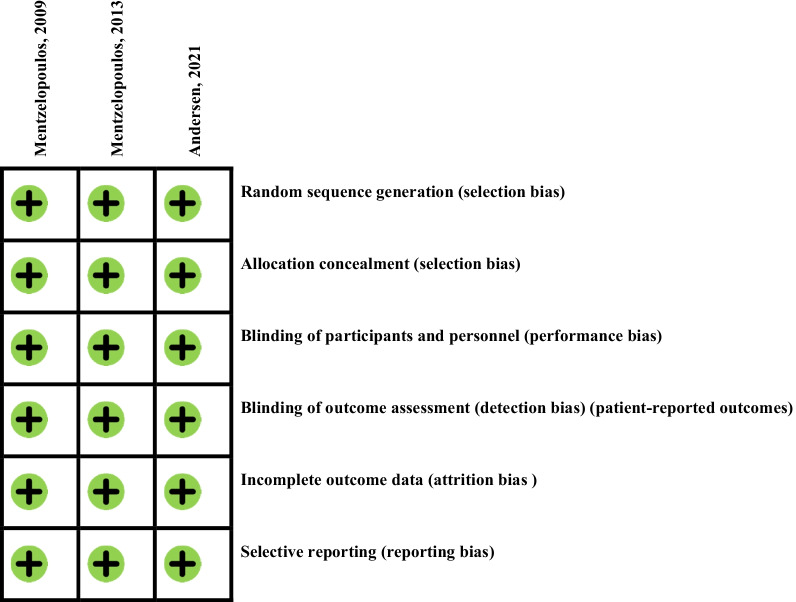
Summary of risk of bias assessment in the reviewed studies

The trials included in the current review were assessed for their quality using Cochrane Collaboration’s tool (Fig. 2). According to the Cochrane Collaboration Risk of Bias assessment, all the trials were classified to have good quality (i.e., low risk of bias for all domains).

### Meta-analysis

#### Survival to hospital discharge

**Fig. 3:**
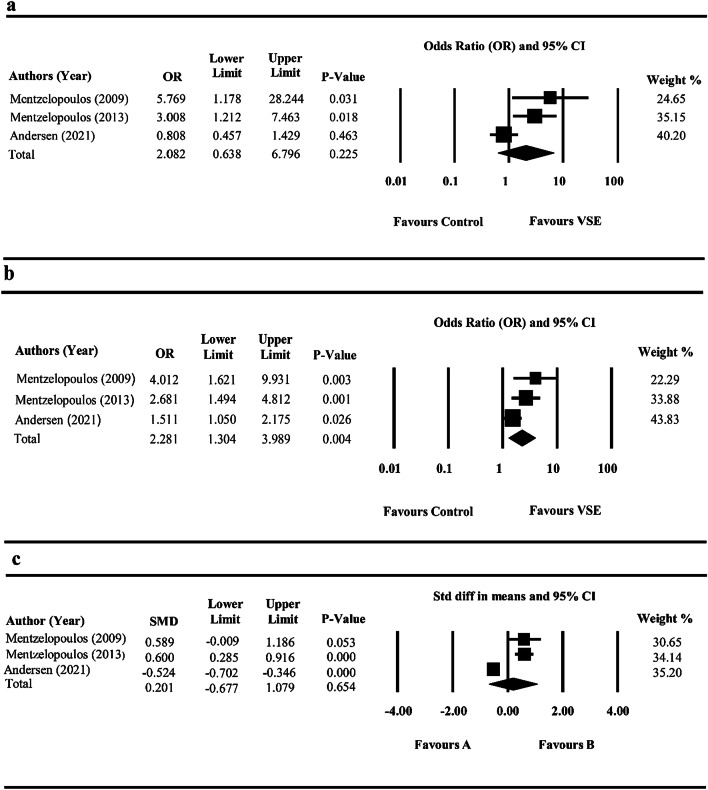
Forest plot illustrating meta-analysis of clinical trials investigated the effect of VSE combination therapy on survival ratio (**a**), ROSC ≥ 15 or 20 min (**b**), and ventilator free days (**c**)

As illustrated in Fig. 3a, combination therapy with VSE could not significantly increase the survival ratio (OR: 2.082, 95% CI: 0.638–6.796, *P *value = 0.225), while the heterogeneity was reported to be high (Cochrane Q test: *I*^2^ = 78.52%, *P *value = 0.010). Sensitivity analysis showed that Andersen et al. study [26] had an impact on the rate of hospital mortality; however, they did not affect the significance of the results (OR: 2.082, 95% CI: 0.638–6.796, *P* value = 0.225).

#### ROSC

The triple VSE combination therapy significantly increased the likelihood of ROSC sustained for at least 15 min (OR: 2.281, 95% CI: 1.304–3.989, *P *value = 0.004) (Fig. 3b) and heterogeneity was not significant (Cochrane *Q* test: *I*^2^ = 63.328, *P *value = 0.065). The results of sensitivity analysis showed that the effect size for the influence of VSE therapy on ROSC was robust after removing studies one by one (OR: 2.281, 95% CI: 1.304–3.989, *P *value = 0.004).

#### MAP during and 15–20 min after CPR

Pool analysis of two studies (368 patients) [27, 28] revealed that VSE combination therapy had a significant effect on MAP during (SMD: 1.069, 95% CI: 0.851–1.288, *P *value < 0.001) and 15–20 min (SMD: 0.831, 95% CI: 0.553–1.110, *P *value < 0.001) after CPR, and no between-study heterogeneity was observed (Cochrane *Q* test: *I*^2^ = 0.00, *P *value = 0.777).

#### Organ failure free days

##### Ventilator free days

The need for mechanical ventilation may indicate respiratory failure. All three studies (869 participants) reported data on ventilator free days [26–28]. The overall meta-analysis showed that there was no significant effect of VSE therapy on ventilator free days, while there was a high between-study heterogeneity (SMD = 0.201, 95% CI: − 0.677 to 1.079 days; *P *value = 0.838; Cochrane *Q* test: *I*^*2*^ = 95.466%; *P *value-heterogeneity < 0.001) (Fig. 3c).

##### Renal failure free days

Two trials including 368 participants reported data on renal failure free days [27, 28]. Meta-analysis of these two studies reported that VSE intervention had a significant effect on the renal failure free days parameter and no between-study heterogeneity was observed (SMD = 0.590; 95% CI: 0.312–0.869 days; *P Value* < 0.001; Cochrane *Q *test: *I*^*2*^ = 0.000%; *P *value-heterogeneity = 0.836).

##### Coagulation failure free days

Another organ failure free days, which was evaluated in two trials (368 participants) [27, 28] was coagulation failure free days. The results of meta-analysis indicated that VSE therapy was associated with a statistically significant reduction in coagulation failure free days, and the analysis had extremely low heterogeneity (SMD = 0.403; 95% CI: 0.128–0.679, *P *value = 0.004; Cochrane *Q* test: *I*^2^ = 0.000%; *P *value heterogeneity = 0.603).

#### Complications

Predefined potential adverse events, including hyperglycemia, pneumonia, electrolyte disturbance, gastrointestinal bleeding, and mesenteric and peripheral ischemia were also evaluated in eligible studies. The risk of adverse events was similar in both groups in all trials.

##### Complications

All three included studies, involving 869 patients assessed insulin requirement. Meta-analysis showed that VSE therapy is associated with a statistically significant higher numbers of patient days with insulin treatment aimed to reduce blood glucose level, and the analysis had extremely low heterogeneity (OR = 1.711; 95% CI: 1.324–2.212, *P* value < 0.001; Cochrane *Q* test: *I*^2^ = 0.000%; *P* value heterogeneity = 0.589).

Publication was not checked, because the asymmetry tests are not valid when the number of studies included in the meta-analysis are few. The summary of Meta-analysis on the effect of VSE triple therapy on different outcomes is illustrated in Table 2 using a random-effect model, based on the type of the study.

**Table 2:** Meta-analysis on the effect of VSE triple therapy on different reported outcomes using a random-effect model

Meta-analysis					Heterogeneity		
Outcomes	N. of studies	N. of participants	ES (95% CI)	*P *value effect	*Q* statistic	*I*-squared (%)	*P *value
Survival	3	869	2.082 (0.638–6.796)	0.225	9.310	78.517	0.010
ROSC	3	869	2.281 (1.304–3.989)	0.004	5.454	63.328	0.065
MAP during CPR	2	368	1.069 (0.851–1.288)	< 0.001	0.080	0.000	0.777
MAP 15–20 min after CPR	2	368	0.831 (0.553–1.110)	< 0.001	0.503	33.446	0.220
Ventilator free days	3	869	0.201 (− 0.677 to 1.079)	0.838	44.108	95.466	< 0.001
Renal failure free days	2	368	0.590 (0.312–0.869)	< 0.001	0.043	0.0001	0.836
Insulin requirement	3	869	1.711 (1.324–2.212)	< 0.001	1.060	0.0001	0.589
Coagulation failure free days	2	368	0.403 (0.128–0.679)	0.004	0.271	0.0001	0.603

*N* number, *ES* effect size, *CI* confidence interval, *ROSC* return of spontaneous circulation, *MAP* mean arterial pressure, *CPR* cardiopulmonary resuscitation

## Discussion

The present systematic review and meta-analysis were performed to investigate the effect of combination therapy with VSE compared to EP plus isotonic saline chloride in patients with cardiac arrest. As presented by the results of the meta-analysis, triple therapy with epinephrine, vasopressin, and steroids significantly increased the likelihood of ROSC sustained for at least 15 min. Although the survival- to- discharge ratio did not achieve a statistical significance between intervention and control groups, but still favored VSE combination group.

ROSC is a sign of a sustained heart rhythm which perfused all body organs after CA. Symptoms of ROSC include a marked respiratory effort, cough, measurable blood pressure, or palpable pulse. Sustained ROSC is regarded when cardiopulmonary resuscitation is halted for at least 15–20 min and the circulation remains stable. Although ROSC is considered a primary outcome in all CA studies, it should be noted that the establishment of ROSC does not necessarily mean the higher survival and favorable outcome of CA victims [34]. Notably, the pathological events in CA are similar to shock syndromes. Similar to shock, failure to take prompt and timely treatment can lead to multiple organ failure (MOF) [35]. Therefore, one of the main outcomes assessed in CA studies is evaluation of MOF, as organ failure free days. Regarding long-term survivors, pool meta-analysis of 368 patients in these RCTs showed a significant increase in renal failure free days, and coagulation failure free days [27, 28]. Regarding the circulatory failure free days and ventilator free days, although the results were not significant, the trend of changes was in favor of the triple VSE combination therapy.

In survivors of CA, severe cerebral disability or vegetative status has the prevalence of 25–50%. Although all three studies evaluated neurological outcome, we could not perform meta-analysis for this variable because of different criteria and scoring scales used in these RCTs. In the study by Andersen et al., neurological status was measured by cerebral performance category (CPC) scale. Favorable neurological outcome was seen in 7.6% of patients in both intervention or control groups on day 30 with no significant difference between the two arms of the trial (*P* value > 0.99) [26]. In the study by Mentzelopoulos et al., the neurologically favorable survival to hospital discharge was assessed. The neurological failure was defined as Glasgow Coma Scale (GCS) < 9. The results of this trial showed a significant improvement in survival to hospital discharge with favorable neurological status [27]. In another study by Mentzelopoulos et al., only neurologic failure was measured as GSC < 9 as part of all-organ failure-free days with favorable results for VSE triple therapy [28].

To the best of our knowledge, no systematic review and extensive meta-analysis has been performed on the effect of VSE triple therapy in CA. Prior reviews have only assessed the effect of one or two drugs (monotherapy or dual therapy) in CA [3, 36–41]. In some recent systematic reviews and meta-analysis, the effect of steroids in CA was evaluated and indicated that steroid use after CA enhances ROSC and survival-to-discharge ratio in the patients with CA [41, 42]. In another meta-analysis, the therapeutic effects of VP were compared with combination therapy using EP and VP. However, this combination was not associated with the improved overall rates of ROSC, long-term survival, or favorable neurological outcomes [37].

The current study is one of few systematic reviews, in which all the included studies were high-quality RCTs and the dose of medications prescribed in the intervention and placebo groups was the same. Herein, three related RCTs were included in the systematic review and a total of 869 subjects were enrolled making the extracted results highly reliable. Given that, EP arm was the same in all the included subjects, the positive results observed in this meta-analysis, the increased ROSC rate appear to be related to administration of VP along with GCs.

Previously, three high-quality population-based cohort studies have shown that steroid supplementation during CPR improves hemodynamic stability, and is associated with high rates of ROSC, survival-to-discharge ratio, and 1-TH survival [43–45]. Mechanism of action of GCs is believed to be via inhibiting free-radical lipid peroxidation, oxidative stress, myocardial apoptosis, cerebral injury, and diminishing overwhelming systemic inflammatory responses,which take place following CA [22, 46, 47]. Furthermore, steroids provide protection against breaking down of endothelial glycocalyx barrier and interstitial swelling [23]. They also help in maintaining cardiovascular stability by preserving myocardial performance, inhibiting catecholamine reuptake, and enhancing vasoconstrictive properties of catecholamines to continue systemic vascular resistance [48]. Moreover, they strengthen contractile responsiveness by adrenergic augmentation [49]. On the other hand, available data have indicated that a low serum cortisol level is associated with unstable hemodynamics after ROSC and less survival rate [50].

The therapeutic effects of VP have been also extensively evaluated in CA. Rationale for using VP is derived from the studies demonstrated a relative deficiency of VP in patients with CA [51]. It is assumed that vasoplegia and pathologic vasodilation following ROSC contribute to a relative VP deficiency [52]. It has been shown that administration of vasopressors leads to an increase in plasma cortisol concentration and better perfusion to adrenal cortex and medulla, which helps in preservation of vascular tone [53].

Following CA, ischemia and damage to all tissues and organs take place, the main of which are brain injury, myocardial dysfunction, ischemia–reperfusion injury, and constant precipitating pathology [54]. These four components can determine the neurohormonal events following CA, even in patients who have rapidly achieved ROSC. Compensatory responses of the patient in face of these pathophysiological processes are aimed to maintain microcirculation, and improve coronary perfusion and contractile function of the heart [2]. Vasoplegia is a pathologic event presented with severe persistent hypotension (MAP < 50 mmHg), and low systemic vascular resistance despite normal or raised cardiac output [55]. It is frequently reported after ischemia–reperfusion syndrome, which itself is a major component of CA [56]. As mentioned, pool analysis of 368 patients [27, 28] revealed that VSE therapy increases MAP significantly during and 15–20 min after CPR.

There have been reports on the depressed VP levels in patients developed vasoplegia following other conditions. Administration of VP has been found to improve coronary and arterial perfusion through mediating P2 purinergic receptors, without causing additional load on the heart [14, 15]. VP induces the secretion of endogenous cortisol and improves vascular response to exogenous catecholamines [57, 58]. Moreover, VP enhances brain and renal vascular perfusion and calcium drive and attenuates the secretion of inflammatory cytokines. It seems that VP could lower mortality rate by improving blood flow to critical organs, decreasing the need for exogenous catecholamines and their subsequent adverse effects [59]. However, despite the presumed efficacy of this agent, the results of two systematic reviews and meta-analyses found no difference between the effects of using VP or norepinephrine on mortality rate in patients with CA [18, 37]. While in septic shock, the results of a meta-analysis indicated that mortality rate was significantly lower in the patients treated with VP or terlipressin compared to norepinephrine [60].

The concern with this combination treatment regimen is the risk of complication and adverse events. The results of the present meta-analysis showed that VSE was associated with a significant increase in insulin requirement. One well- known adverse effects of glucocorticoids is hyperglycemia, and a safe way for its management in hospitalized patients is administration of insulin. It is noteworthy that VSE patients, despite receiving more insulin, did not experience more frequent episodes of hyperglycemia. Regarding other potential adverse effects and possible complications, no significant difference was observed between the two study groups, including pneumonia.

### Limitations of the study

Clinical trials are among the studies with the highest quality and most reliability and all the studies enrolled in this meta-analysis were clinical trials. Although, the enrolled RCTs were judged to have high quality, the results should be interpreted with caution. One major limitation of the included RCTs of this meta-analysis was comparison of three interventions simultaneously, making it difficult to discern which of the agents in VSE combination therapy exerts the positive effects observed in this population of the patients. Furthermore, it is yet indefinite whether each of these interventions alone can have beneficial effects on the patients with CA or VSE combination therapy has synergistic properties. Moreover, it is unclear whether the observed beneficial effects are due to the physiological effects of these drugs or a reduction in the need to exogenous vasopressors and their subsequent side effects; a case reported in Mentzelopoulos’ trial on 2009 which indicated that the use of this combination in 60-day follow-up significantly reduced the requirement for vasopressor [28]. If post-arrest myocardial function and physiological parameters were measured at different post-resuscitation times in these studies, then the results would be more reliable and give us a better overview of the exact process happening at neurohormonal level in CA. The last limitation was that, in spite of wide systematic search, a few relevant studies were eligible for inclusion in this meta-analysis.

## Conclusion and the future prospects

The present study was the first investigation systematically reviewed the clinical studies on VSE triple therapy in CA. It was tried to cover all the existing literature in this field. Fortunately, dispersion of studies in this area was not high, they had similar design and they included common outcomes. Herein, three relatively uniform RCTs with high quality and low risk of bias were included. To the best of our knowledge, no systematic review and extensive meta-analysis have been recently performed on the effect of VSE triple therapy in CA.

The results of the current systematic review and meta-analysis indicated that triple therapy with EP, VP, and steroids compared to EP plus isotonic saline chloride significantly increased the likelihood of ROSC sustained for at least 15 min. Although the survival- to- discharge ratio did not achieve a statistical significance between intervention and control groups, but still favored VSE combination group.

For the future studies, it is recommended to determine the exact role of each component of this intervention and also who would be likely to benefit most from VSE combination therapy. Furthermore, it is suggested to explore the optimal dosage and duration of treatment with VSE during resuscitation and after achievement of ROSC. Moreover, the long-term influence of VSE combination therapy on post-arrest myocardial function, physiological parameters, and neurological outcomes of these patients should be studied in the future investigations.

## Supplementary Information

**Additional file 1:** Search strategy of the literature.

## Data Availability

The datasets used and/or analysed during the current study available from the corresponding author on reasonable request.

## Abbreviations

VSE: Vasopressin, steroid, and epinephrine
CA: Cardiac arrest
MAP: Mean arterial pressure
ROSC: Return of spontaneous circulation
ACLS: Advanced cardiac life support
BLS: Basic life support
CPR: Cardiopulmonary resuscitation
EP: Epinephrine
VP: Vasopressin
OTR: Oxytocin receptors
V1R: V1 vascular receptors
RCT: Randomized clinical trial
OHCA: Out-of-hospital cardiac arrest
GCs: Glucocorticoids
PCAS: Post-cardiac arrest syndrome
IHCA: In-hospital cardiac arrest
MOF: Multiple organ failure
CPC: Cerebral performance category
GCS: Glasgow Coma Scale
OR: Odds ratio
CI: Confidence interval
SMD: Standardized mean difference
