# Supplementary material for: Efficacy of combination triple therapy with vasopressin, steroid, and epinephrine in cardiac arrest: a systematic review and meta-analysis of randomized-controlled trials
Source: J Intensive Care. 2022 Feb 2;10:5. doi: 10.1186/s40560-022-00597-5 (PMC8809021; doi:10.1186/s40560-022-00597-5)
Supplement: Supplementary file 1 — Additional file 1: Search strategy of the literature. [file 40560_2022_597_MOESM1_ESM.docx]

**PubMed:**

**(hydrocortisone[Mesh] OR hydrocortisone[tiab] OR "hydrocortisone sodium phosphate" [Supplementary Concept] OR Cortisol*[tiab] OR steroid*[Mesh] OR steroid*[tiab] OR "catatoxic steroids"[tiab] OR Glucocorticoids[Mesh] OR Glucocorticoid*[tiab] OR Glucocorticoids[tiab] OR Corticosteroid[tiab] OR "alclometasone dipropionate"[tiab] OR amcinonide[tiab] OR Beclomethasone*[tiab] OR Budesonide[tiab] OR ciclesonide[tiab] OR Clobetasol[tiab] OR "clobetasone butyrate"[tiab] OR clocortolone[tiab] OR "clocortolone pivalate"[tiab] OR Desoximetasone[tiab] OR Dexamethasone*[tiab] OR "dichlorisone acetate"[tiab] OR diflorasone[tiab] OR Diflucortolone[tiab] OR difluprednate[tiab] OR "drocinonide phosphate potassium"[tiab] OR flumethasone*[tiab] OR "Fluocinolone Acetonide"[ tiab] OR Fluocinonide[tiab] OR "fluocortin butyl ester"[ tiab] OR Fluocortolone[tiab] OR Fluorometholone[tiab] OR Fluprednisolone[tiab] OR Flurandrenolone[tiab] OR "Fluticasone"[tiab] OR halometasone[tiab] OR medrysone[tiab] OR "Melengestrol Acetate"[tiab] OR ethylprednisolone[tiab] OR "Methylprednisolone Hemisuccinate"[tiab] OR Methylprednisolone[tiab] OR Paramethasone[tiab] OR prednicarbate[tiab] OR prednisolone*[tiab] OR prednisone[tiab] OR rimexolone[tiab] OR Triamcinolone*[tiab]** OR betamethasone[tiab] OR cortisone*[tiab] OR Fludrocortisone[tiab] OR fluocinolone[tiab] OR fluticasone[tiab] OR mometasone[tiab] OR glucocorticosteroid[tiab] OR mineralocorticoid[tiab]**) AND ("Epinephrine"[Mesh] OR "Epinephrine"[tiab] OR "Adrenaline"[tiab] OR "4-(1-Hydroxy-2-(methylamino)ethyl)-1,2-benzenediol"[tiab] OR "Epinephrine Hydrochloride"[tiab] OR "Adrenaline Hydrochloride"[tiab] OR "Epinephrine Bitartrate"[tiab] OR " Adrenaline Acid Tartrate"[tiab] OR " Adrenaline Bitartrate"[tiab]) AND ( "Vasopressin" OR "beta Hypophamine "[tiab] OR "beta-Hypophamine"[ tiab] OR " Antidiuretic Hormones "[tiab] OR " Pitressin "[tiab]) AND ("Heart Arrest"[Mesh] OR "Out-of-Hospital Cardiac Arrest"[Mesh] OR "Heart Arrest, Induced"[Mesh] OR "Out of Hospital Cardiac Arrests"[tiab] OR "Heart stop"[tiab] OR "Sudden Cardiac Death"[tiab] OR ''cardiac arrest''[tiab] OR ''heart arrest''[tiab] OR ''cardiac attack''[tiab] OR ''heart attack''[tiab]) AND (Survival[Mesh] OR Survival*[tiab] OR Mortality[Mesh] OR Mortality[tiab] OR Mortalities[tiab] OR "Case Fatality Rate*" [tiab] OR "Death Rate*"[tiab] OR "Mortality Rate*"[tiab] OR "Survival Rate"[Mesh] OR "Treatment Outcome"[Mesh] OR Survival[tiab] OR "survival to admission"[tiab] OR "survival to discharge"[tiab] OR "one year survival rate*"[tiab] OR "hospital discharge"[tiab] OR "clinical outcome"[tiab] OR "recovery rate"[tiab] OR outcome[tiab]) NOT (rats[mesh] OR rat[tiab] OR mice[mesh] OR swine[mesh] OR mouse[tiab] OR mice[tiab] OR in-vitro[tiab] OR "in vitro"[tiab] OR Pig[tiab] OR Rabbit*[tiab] OR Rooster*[tiab] OR cell[tiab] OR cow*[tiab] OR suide[tiab] OR warthogs[tiab] OR broiler*[tiab] OR "in vivo"[tiab] OR in-vivo[tiab] OR "in ovo"[tiab] OR in-ovo[tiab] OR infant*[tiab] OR infant[mesh] OR genes[mesh] OR gene[tiab] OR cistron*[tiab] OR genetic*[tiab] OR fishes[mesh] OR Fish*[tiab] OR Horses[mesh] OR horse*[tiab] OR Pregnancy[Mesh] OR Pregnanc*[tiab] OR Gestation[tiab] OR nursing[tiab] OR pregnant*[tiab] OR "Breast Feeding"[Mesh] OR "Breast Feeding"[tiab] OR Breastfeeding[tiab] OR "Bottle Feeding"[Mesh] OR "Bottle feed"[tiab] OR Bottle-feed[tiab] OR Breast-Feeding [tiab] OR "Bottle Feeding"[tiab] OR "Breast Milk"[tiab] OR "Human Milk"[tiab] OR protein[tiab] OR goat*[tiab] OR chicken[tiab] OR shrimp*[tiab] OR sheep*[tiab] OR crab*[tiab] OR monkey*[tiab] OR rabbit*[tiab] OR duck*[tiab] OR fowl*[tiab] OR roots[tiab] OR bacteria*[tiab] OR camel*[tiab])**

**Scopus:**

**TITLE-ABS-KEY(hydrocortisone) OR TITLE-ABS-KEY(Cortisol*) OR TITLE-ABS-KEY(steroid*) OR TITLE-ABS-KEY(Glucocorticoid*) OR TITLE-ABS-KEY(Corticosteroid) OR TITLE-ABS-KEY(alclometasone dipropionate) OR TITLE-ABS-KEY(amcinonide) OR TITLE-ABS-KEY(Beclomethasone*) OR TITLE-ABS-KEY(Budesonide) OR TITLE-ABS-KEY(ciclesonide) OR TITLE-ABS-KEY(Clobetasol) OR TITLE-ABS-KEY(clocortolone) OR TITLE-ABS-KEY(Desoximetasone*) OR TITLE-ABS-KEY(diflorasone) OR TITLE-ABS-KEY(Diflucortolone) OR TITLE-ABS-KEY(difluprednate) OR TITLE-ABS-KEY(flumethasone*) OR TITLE-ABS-KEY(Fluocinonide) OR TITLE-ABS-KEY(Fluocortolone) OR TITLE-ABS-KEY(Fluorometholone) OR TITLE-ABS-KEY(Fluticasone) OR TITLE-ABS-KEY(halometasone) OR TITLE-ABS-KEY(medrysone) OR TITLE-ABS-KEY(ethylprednisolone) OR TITLE-ABS-KEY(Paramethasone) OR TITLE-ABS-KEY(prednicarbate) OR TITLE-ABS-KEY(prednisolone*) OR TITLE-ABS-KEY(prednisone) OR TITLE-ABS-KEY(rimexolone) OR TITLE-ABS-KEY(Triamcinolone) OR** TITLE-ABS-KEY(betamethasone) OR TITLE-ABS-KEY(cortisone*) OR TITLE-ABS-KEY(Fludrocortisone) OR TITLE-ABS-KEY(fluocinolone) OR TITLE-ABS-KEY(fluticasone) OR TITLE-ABS-KEY(mometasone) OR TITLE-ABS-KEY(glucocorticosteroid) OR TITLE-ABS-KEY(mineralocorticoid) **AND (TITLE-ABS-KEY (Epinephrine) OR TITLE-ABS-KEY (Adrenaline) OR TITLE-ABS-KEY (EpinephrineAcetate) OR TITLE-ABS-KEY (4-1-Hydroxy-2-methylaminoethyl-1,2-benzenediol) OR TITLE-ABS-KEY (EpinephrineHydrochloride) OR TITLE-ABS-KEY (AdrenalineHydrochloride) OR TITLE-ABS-KEY (EpinephrineBitartrate) OR TITLE-ABS-KEY ( AdrenalineAcid Tartrate) OR TITLE-ABS-KEY (AdrenalineBitartrate)) AND (TITLE-ABS-KEY (Vasopressin) OR TITLE-ABS-KEY ( betaHypophamine) OR TITLE-ABS-KEY (beta-Hypophamine) OR TITLE-ABS-KEY (AntidiureticHormones) OR TITLE-ABS-KEY (Pitressin )) AND (TITLE-ABS-KEY(Heart Arrest) OR TITLE-ABS-KEY(cardiac arrest) OR TITLE-ABS-KEY(heart arrest) OR TITLE-ABS-KEY(cardiac attack) OR TITLE-ABS-KEY(heart attack) OR TITLE-ABS-KEY(Out-of-Hospital Cardiac Arrest) OR TITLE-ABS-KEY(Heart Arrest, Induced) OR TITLE-ABS-KEY(Out of Hospital Heart Arrests) OR TITLE-ABS-KEY(Out of Medical Institution Heart Arrests) OR TITLE-ABS-KEY(Out of Medical Centre Heart Arrests) OR TITLE-ABS-KEY(Out of Medical Center Heart Arrests) OR TITLE-ABS-KEY(Out of health center Heart Arrests) OR TITLE-ABS-KEY(Out of health center Heart Arrests) OR TITLE-ABS-KEY(Out of Clinic Heart Arrests) OR TITLE-ABS-KEY(Out of lazaretto Heart Arrests) OR TITLE-ABS-KEY(Out of hospice Heart Arrests) OR TITLE-ABS-KEY(Out of health center Heart Arrests) OR TITLE-ABS-KEY(Out of Hospital Cardiac Arrests) OR TITLE-ABS-KEY(Out of Medical Institution Cardiac Arrests) OR TITLE-ABS-KEY(Out of Medical Centre Cardiac Arrests) OR TITLE-ABS-KEY(Out of Medical Center Cardiac Arrests) OR TITLE-ABS-KEY(Out of health center Cardiac Arrests) OR TITLE-ABS-KEY(Out of health center Cardiac Arrests) OR TITLE-ABS-KEY(Out of Clinic Cardiac Arrests) OR TITLE-ABS-KEY(Out of lazaretto Cardiac Arrests) OR TITLE-ABS-KEY(Out of hospice Cardiac Arrests) OR TITLE-ABS-KEY(Out of health center Cardiac Arrests) OR TITLE-ABS-KEY(Out of Hospital Cardiac Arrests) OR TITLE-ABS-KEY(Cardiopulmonary Arrest) OR TITLE-ABS-KEY(Induced Cardiac Arrest) OR TITLE-ABS-KEY(Induced Heart Arrest) OR TITLE-ABS-KEY(Heart halt) OR TITLE-ABS-KEY(Heart stop) OR TITLE-ABS-KEY(Sudden Cardiac Death)) AND (TITLE-ABS-KEY(Survival*) OR TITLE-ABS-KEY(Mortality) OR TITLE-ABS-KEY(Mortalities) OR TITLE-ABS-KEY(CaseFatality Rate*) OR TITLE-ABS-KEY(DeathRate*) OR TITLE-ABS-KEY(MortalityRate*) OR TITLE-ABS-KEY(SurvivalRate) OR TITLE-ABS-KEY(TreatmentOutcome) OR TITLE-ABS-KEY(survivaltoadmission) OR TITLE-ABS-KEY(hospitaldischarge) OR TITLE-ABS-KEY(clinicaloutcome) OR TITLE-ABS-KEY(recoveryrate) OR TITLE-ABS-KEY(outcome)) AND NOT (TITLE-ABS-KEY(rats) OR TITLE-ABS-KEY(rat) OR TITLE-ABS-KEY(mice) OR TITLE-ABS-KEY(swine*) OR TITLE-ABS-KEY(mouse) OR TITLE-ABS-KEY(in-vitro) OR TITLE-ABS-KEY(invitro) OR TITLE-ABS-KEY(Pig) OR TITLE-ABS-KEY(Rabbit*) OR TITLE-ABS-KEY(Rooster*) OR TITLE-ABS-KEY(cell) OR TITLE-ABS-KEY(cow*) OR TITLE-ABS-KEY(suide) OR TITLE-ABS-KEY(warthogs) OR TITLE-ABS-KEY(broiler) OR TITLE-ABS-KEY(in vivo) OR TITLE-ABS-KEY(in-vivo) OR TITLE-ABS-KEY(in-ovo) OR TITLE-ABS-KEY(infant*) OR TITLE-ABS-KEY(gene*) OR TITLE-ABS-KEY(cistron*) OR TITLE-ABS-KEY(genetic*) OR TITLE-ABS-KEY(Fish*) OR TITLE-ABS-KEY(horse*) OR TITLE-ABS-KEY(Pregnanc*) OR TITLE-ABS-KEY(Gestation) OR TITLE-ABS-KEY(nursing) OR TITLE-ABS-KEY(pregnant*) OR TITLE-ABS-KEY(Breast Feeding) OR TITLE-ABS-KEY(Breastfeeding) OR TITLE-ABS-KEY(Bottle Feeding) OR TITLE-ABS-KEY(Bottle feed) OR TITLE-ABS-KEY(Bottle-feed) OR TITLE-ABS-KEY(BreastMilk*) OR TITLE-ABS-KEY(HumanMilk*) OR TITLE-ABS-KEY(protein) OR TITLE-ABS-KEY(goat*) OR TITLE-ABS-KEY(chicken) OR TITLE-ABS-KEY(shrimp*) OR TITLE-ABS-KEY(sheep*) OR TITLE-ABS-KEY(cat) OR TITLE-ABS-KEY(dog*) OR TITLE-ABS-KEY(crab*) OR TITLE-ABS-KEY(monkey*) OR TITLE-ABS-KEY(rabbit*) OR TITLE-ABS-KEY(duck*) OR TITLE-ABS-KEY(fowl*) OR TITLE-ABS-KEY(roots) OR TITLE-ABS-KEY(bacteria*) OR TITLE-ABS-KEY(camel*))**

# Web of Science:

#

# (TS=(hydrocortisone) OR TS=("hydrocortisone sodium phosphate") OR TS=(Cortisol*) OR TS=(steroid*) OR TS=(Glucocorticoid*) OR TS=(Corticosteroid) OR TS=(amcinonide) OR TS=(Beclomethasone*) OR TS=(Budesonide) OR TS=(Clobetasol) OR TS=(clocortolone) OR TS=(Desoximetasone*) OR TS=("ichlorisoneacetate") OR TS=(diflorasone) OR TS=(Diflucortolone) OR TS=(flumethasone*) OR TS=("FluocinoloneAcetonide") OR TS=(Fluocinonide) OR TS=(Fluocortolone) OR TS=(Fluorometholone) OR TS=(Flurandrenolone) OR TS=(Fluticasone) OR TS=(halometasone) OR TS=(medrysone) OR TS=(Melengestrol Acetate) OR TS=(ethylprednisolone) OR TS=("MethylprednisoloneHemisuccinate") OR TS=(Paramethasone) OR TS=(prednicarbate) OR TS=(prednisolone*) OR TS=(prednisone) OR TS=(rimexolone) OR TS=(Triamcinolone) OR TS=(betamethasone) OR TS=(cortisone*) OR TS=(Fludrocortisone) OR TS=(fluocinolone) OR TS=(fluticasone) OR TS=(mometasone) OR TS=(glucocorticosteroid*) OR TS=(mineralocorticoid))AND (TS=(Epinephrine) OR TS=(Adrenaline) OR TS=( Epinephrine Acetate) OR TS=(4-(1-Hydroxy-2-(methylamino)ethyl)-1,2-benzenediol) OR TS=(Epinephrine Hydrochloride) OR TS=(Adrenaline Hydrochloride) OR TS=( Epinephrine Bitartrate) OR TS=(Adrenaline Acid Tartrate) OR TS=( Adrenaline Bitartrate)) AND (TS=(Vasopressin) OR TS=( beta Hypophamine ) OR TS=(beta-Hypophamine) OR TS=( Antidiuretic Hormones) OR TS=( Pitressin )) AND (TS=("Heart Arrest") OR TS=("cardiac arrest") OR TS=("heart arrest") OR TS=("cardiac attack") OR TS=("heart attack") OR TS=("Out-of-Hospital Cardiac Arrest") OR TS=("Heart Arrest, Induced") OR TS=("Out of Hospital Heart Arrests") OR TS=("Out of Medical Institution Heart Arrests") OR TS=("Out of Medical Centre Heart Arrests") OR TS=("Out of Medical Center Heart Arrests") OR TS=("Out of health center Heart Arrests") OR TS=("Out of Clinic Heart Arrests") OR TS=("Out of lazaretto Heart Arrests") OR TS=("Out of hospice Heart Arrests") OR TS=("Out of Hospital Cardiac Arrests") OR TS=("Out of Medical Institution Cardiac Arrests") OR TS=("Out of Medical Centre Cardiac Arrests") OR TS=("Out of Medical Center Cardiac Arrests") OR TS=("Out of health center Cardiac Arrests") OR TS=("Out of health center Cardiac Arrests") OR TS=("Out of Clinic Cardiac Arrests") OR TS=("Out of lazaretto Cardiac Arrests") OR TS=("Out of hospice Cardiac Arrests") OR TS=("Out of health center Cardiac Arrests") OR TS=("Out of Hospital Cardiac Arrests") OR TS=("Cardiopulmonary Arrest") OR TS=("Induced Cardiac Arrest") OR TS=("Induced Heart Arrest") OR TS=("Heart halt") OR TS=("Heart stop") OR TS=("Sudden Cardiac Death")) AND (TS=(Survival*) OR TS=(Mortality) OR TS=(Mortalities) OR TS=("Case Fatality Rate*") OR TS=("Death Rate*") OR TS=("Mortality Rate*") OR TS=("Survival Rate") OR TS=("Treatment Outcome") OR TS=("survival to admission") OR TS=("one year survival rate") OR TS=("hospital discharge") OR TS=("clinical outcome") OR TS=("recovery rate") OR TS=(outcome)) NOT (TS=(rats) OR TS=(rat) OR TS=(mice) OR TS=(swine*) OR TS=(mouse) OR TS=(in-vitro) OR TS=("in vitro") OR TS=(Pig) OR TS=(Rabbit*) OR TS=(Rooster*) OR TS=(cell) OR TS=(cow*) OR TS=(suide) OR TS=(warthogs) OR TS=(broiler) OR TS=("in vivo") OR TS=(in-vivo) OR TS=("in ovo") OR TS=(in-ovo) OR TS=(infant*) OR TS=(gene*) OR TS=(cistron*) OR TS=(genetic*) OR TS=(Fish*) OR TS=(horse*) OR TS=(Pregnanc*) OR TS=(Gestation) OR TS=(nursing) OR TS=(pregnant*) OR TS=("Breast Feeding") OR TS=(Breastfeeding) OR TS=("Bottle Feeding") OR TS=("Bottle feed") OR TS=(Bottle-feed) OR TS=("Breast Milk") OR TS=("Human Milk*") OR TS=(protein) OR TS=(goat*) OR TS=(chicken) OR TS=(shrimp*) OR TS=(sheep*) OR TS=(cat) OR TS=(dog*) OR TS=(crab*) OR TS=(monkey*) OR TS=(rabbit*) OR TS=(duck*) OR TS=(fowl*) OR TS=(roots) OR TS=(bacteria*) OR TS=(camel*))

**Results (5 October 2021):**

**PubMed=14**

**Scopus=27**

**ISI=12**

**SUM=53**
